# Evaluation of postoperative bleeding risk after dental extractions in patients on antithrombotic medication: A comparison of machine learning and clinical experience

**DOI:** 10.1007/s00784-025-06590-0

**Published:** 2025-10-27

**Authors:** Marie Sophie Katz, Orian Nathan Mahlow, Rajae Benidamou, Mark Ooms, Marius Heitzer, Dirk Elvers, Frank Hölzle, Ali Modabber

**Affiliations:** 1 Department of Oral and Maxillofacial Surgery, University Hospital RWTH Aachen, Pauwelsstraße 30, 52074 Aachen, Germany; 2 Chair of Electronic Commerce, Goethe University Frankfurt, Theodor-W.-Adorno-Platz 4, 60323 Frankfurt am Main, Germany

**Keywords:** Postoperative bleeding, Antiplatelet therapy, Oral anticoagulation, Dental extraction, Machine learning algorithm

## Abstract

**Objectives:**

The aim of this study was to identify high-risk dental extractions in patients taking antiplatelet (AP) medication or anticoagulants (ACs) and to compare an experienced surgeon’s decisions with machine learning (ML) algorithms.

**Materials and methods:**

Our study included 2000 procedures, of which 1788 were conducted in patients under monotherapy with AP medication, vitamin K antagonists (VKAs), heparin, or direct oral anticoagulants (DOACs), 426 were performed under dual therapy, and 27 under triple therapy. Four algorithms, logistic regression (LR), eXtreme gradient boost (XGB), random forest (RF), and K-nearest neighbors (KNN), were trained with 80% (1600 procedures) of the derived data. Afterwards, an experienced oral surgeon and the algorithms were tested on the remaining 20% (400 procedures) of the data to evaluate the predictive power with respect to bleeding incidents.

**Results:**

The incidence of hemorrhagic events was low (4.35%). Dual anticoagulation significantly affected the risk of bleeding. Evaluating the results of the predictions, all four algorithms outperformed the surgeon in terms of balanced accuracy (LR: 58%; RF: 59%; XGB: 61%; KNN: 62%; surgeon: 53%).

**Conclusions:**

Decision-making based on various parameters influencing bleeding risk is complex, and surgeons tend to overestimate this risk. Both the algorithms and the surgeon had a share of false positive predictions; however, in a medical context, preventive overcaution does less damage than underestimation.

**Clinical relevance:**

Algorithms can provide an objective assessment of bleeding risk and help determine risk profiles, uncover variables with the highest predictive power, and serve as guidance on postoperative observation periods.

**Trial registration:**

This study was approved by the Ethics Committee of the Medical Faculty of RWTH Aachen (Decision Number 24–353). This was a retrospective clinical study designed to analyze postoperative bleeding after dental extractions in patients under antithrombotic medication and to evaluate the prediction of bleeding events by different algorithms and human experience.

**Supplementary Information:**

The online version contains supplementary material available at 10.1007/s00784-025-06590-0.

## Objectives

 A growing share of patients is taking antithrombotic medication, and the impact of the various medications on the risk of postoperative hemorrhage after oral surgery is sometimes difficult to anticipate, even for experienced surgeons [1–4].

Bleeding risk in patients under antiplatelet (AP) medication and anticoagulants (ACs) has been analyzed in previous studies, ranking operations on patients under monotherapy with a lower risk than procedures under dual therapy [3, 5–7]. AP medication is usually associated with less postoperative bleeding than vitamin K antagonists (VKAs) or direct oral anticoagulants (DOACs) [3, 8]. However, not only does the drug itself influence bleeding risk, but also the extent of the surgery has a significant influence on postoperative incidents: Ueda et al. found that patients with osteotomies, vertical incisions, and posterior or multiple extractions have a higher risk of bleeding episodes [9].

Some attempts to predict bleeding in patients receiving antithrombotic medication have been made, such as the HAS-BLED score, which is also used in other medical fields, but this score was found to be insufficient for dental extractions in patients taking warfarin by Kataoka et al. [10]. A previous study showed that patients under double anticoagulation therapy benefit especially from a prolonged observation period, but that most patients who were preventively administered inpatient care because of their bleeding risk did not bleed at all [3]. This indicates that surgeons often overcautiously administer inpatient care. Identifying bleeding risks is highly complex because it is influenced by a broad range of factors, such as osteotomies, the number of teeth that should be removed, medications, and many more. Given the long list of factors that may have an influence, it is hard to accurately weight their relative importance.

Algorithms based on machine learning (ML) and neural networks are increasingly used in dentistry and have been proven to be helpful in various studies, such as sex determination based on dentition [11], dental shade matching [12], implant placement [13], detection of early childhood caries [14, 15], and predicting tooth extraction based on X-rays [16]. As algorithms provide objective decisions based on data they are given to train on, they can in some cases be more efficient and more precise than human experience and clinical heuristics [13, 16]. A sufficient sample size is necessary, especially if different parameters are involved, such as different kinds of medications and procedures, and if the required effect, such as bleeding risk, is considered low [8].

Various kinds of ML algorithms can be applied as prediction models: Logistic regression (LR) is a basic statistical method for predicting a binary dependent variable [17]; random forest (RF) is an ML algorithm based on the construction of multiple decision trees during training that outputs the class with the majority vote or the average prediction from all individual trees, aiming to improve accuracy and reduce overfitting [14, 18]; eXtreme gradient boost (XGB) is a gradient-boosting ML algorithm that iteratively combines weak learners (e.g., decision trees) to improve overall performance by minimizing prediction errors [12, 19]; and K-nearest neighbors (KNN) is a non-parametric learning method that classifies a data point by looking at the “k” training examples closest to it and assigning the most common label among them, making decisions based on local patterns and similarities in the data [11].

The aim of this study was to evaluate whether ML algorithms are better at identifying patients receiving antithrombotic medication who experienced postoperative bleeding episodes than an experienced oral surgeon.

## Methods

### Study design

This study was approved by the local Clinical Research Ethics Committee (Decision Number EK 24–353).

It aimed to identify postoperative bleeding episodes following dental extractions in patients under AP or AC therapy, using ML algorithms based on clinical data obtained through a centralized retrospective chart review.

## Sample size calculation

The existing literature on clinical studies on postoperative bleeding after dental extractions in patients with antithrombotic medication was reviewed to calculate a suitable range for the sample size and define the investigation period. The required sample size was calculated based the study by Yagyuu et al., who found a post-extraction bleeding incidence of 10.4% in DOAC patients and 12% in patients under VKA in a cohort of 541 patients who underwent 634 procedures [20]. The incidence of postoperative bleeding was the primary outcome considered when calculating the sample size. The statistical program G* Power Version 3.1.9.6 (Heinrich-Heine-Universität, Düsseldorf, Germany) was used for the calculation, with an alpha value of 0.05, an effect size of 0.1, and a statistical power of 95%. Based on these parameters, a sample size of at least 1135 procedures was found suitable to test the null hypothesis that there is no significant difference concerning postoperative bleeding events, with 95% power and a 95% confidence interval. As the entire patient cohort that we analyzed was also treated with gelatin sponges, which can lower the incidence of postoperative bleeding [21], we decided to collect a dataset of 2000 procedures.

## Data collection

The data of the procedures of patients who underwent dental extraction under AP or AC therapy between January 2014 and August 2024 in our university hospital department were collected.

The exclusion criteria were procedures involving underaged patients (< 18 years), patients without any antithrombotic medication, patients with inherited bleeding disorders, incomplete postoperative documentation (defined as missing essential information regarding postoperative bleeding events or follow-up within the first 10 days after extraction), and procedures including intraoral operations other than dental extractions (e.g., biopsies and root resections).

In all procedures, a standardized local hemostatic protocol was followed, including the application of a gelatin sponge (Gelastypt^®^, Sanofi-Aventis, Frankfurt am Main, Germany) to the alveolus after extraction, followed by suturing with a 4 − 0 Vicryl^®^ suture (Ethicon Inc, Cincinnati, OH, USA).

The following data were derived from the medical records: patient sex, patient age at the time of procedure, type of medication, number of teeth extracted and method of removal (extraction or osteotomy), region of surgery (anterior, posterior, or both), and incidence of bleeding.

The number of teeth extracted per procedure varied depending on the patient’s general health and dental condition. Typically, no more than 10 teeth were removed in a single session to reduce surgical burden and facilitate healing. In cases requiring extraction of more teeth, treatment was divided into multiple sessions accordingly.

Postoperative bleeding was defined as any documented bleeding event occurring after the extraction procedure and requiring clinical attention, irrespective of severity. Due to the low number of severe cases requiring additional intervention (e.g., suturing), no further grading by severity was applied.

### Statistical analysis

For the descriptive statistics of the categorical data (sex, location of surgery, and incidence of postoperative bleeding), the differences between the groups were analyzed using a Chi-square test or a Fisher’s Exact test. The continuous data (age and number of teeth removed by extraction or osteotomy) were evaluated using the Wilcoxon-Mann–Whitney test, as they lacked a Gaussian distribution according to the Shapiro–Wilk test. P-values < 0.05 were considered significant.

**Fig. 1:**
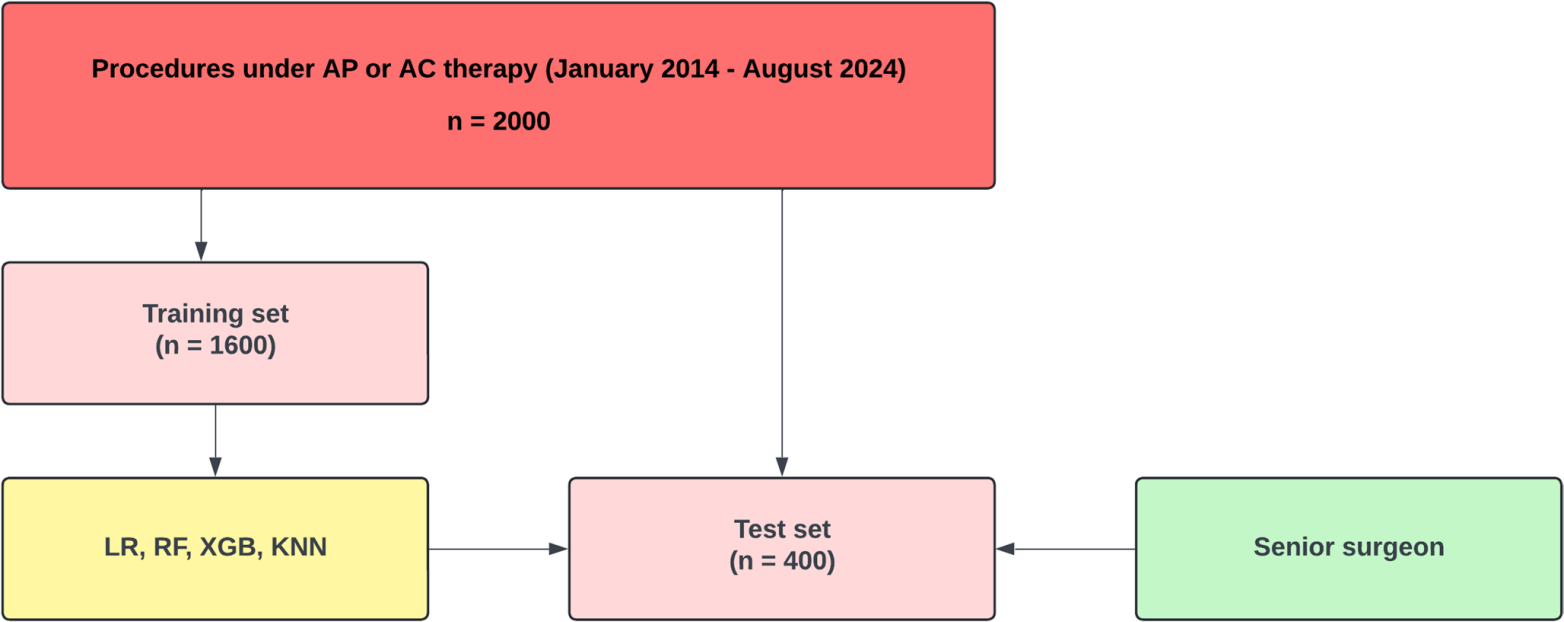
Machine learning workflow and evaluation process

The data were then randomly divided into a training set (80%; 1600 procedures) and a test set (20%; 400 procedures), and all four algorithms (LR, RF, XGB, and KNN) were trained on the training set first. Afterwards, all four algorithms separately evaluated the test set, and a senior oral surgeon with over 15 years of experience was asked to assess the same test set. Afterwards, the prediction accuracy of the algorithms and the surgeon were compared (Fig. 1).

The dataset was randomly divided into a training set (80%; 1600 procedures) and a test set (20%; 400 procedures). Four algorithms—Logistic Regression (LR), Random Forest (RF), eXtreme Gradient Boosting (XGB), and K-Nearest Neighbors (KNN)—were trained on the training set and subsequently used to generate predictions on the test set. A senior oral surgeon with over 15 years of experience also assessed the same test set. The prediction performance of the algorithms and the surgeon was then compared.

## Results

Our final study sample comprised 2000 surgeries, including 1788 operations under monotherapy with AP medication (670), VKA (328), subcutaneous low molecular weight heparin (LMWH) (184), intravenous unfractionated heparin (UFH) (49), or DOAC (547), 213 under dual therapy, and 9 procedures under triple therapy.

The mean age of the patients was 68.5 years; 695 procedures were performed on female patients (34.7%), and 1305 procedures were carried out on male patients (65.3%). A total of 7125 teeth were removed, of which 6607 needed simple extraction, and 518 required osteotomies. Of the procedures, 256 included only removal of anterior teeth, 1009 included only posterior teeth, and 735 involved both regions. In total, 87 postoperative bleeding events were recorded (4.35%).

Comparing the procedures through basic descriptive statistical tests, only dual therapy had a significant influence on bleeding risk (*p* < 0.001), while different kinds of monotherapy, triple therapy, sex, age, location of the surgery, and the number of teeth removed by extraction and osteotomy did not differ significantly (*p* = 0.072, *p* = 1.000, *p* = 0.251, *p* = 0.361, *p* = 0.735, *p* = 0.376, *p* = 0.359, respectively) (Table 1).

**Table 1:** Procedure characteristics and bleeding events

		No bleeding events	Bleeding events	Total	*P*-value
Type of medication					
Monotherapy	**AP** ^**a**^	651	19	670	0.072
	**VKA** ^**b**^	316	12	328	
	**Heparin** ^**c**^	227	6	233	
	**DOAC** ^**d**^	517	30	547	
Dual therapy		193	20	213	< 0.001*
Triple therapy		9	0	9	1.000
Sex					
Male		1243	62	1305	0.251
Female		670	25	695	
Mean Age					
		68.4 (SD ± 13.5)	69.7 (SD ± 13.9)	68.5 (SD ± 13.5)	0.361
Location					
Only anterior teeth		247	9	265	0.735
Only posterior teeth		965	44	1009	
Involving anterior and posterior teeth		701	34	735	
Mean number of teeth removed					
By extraction		3.3 (SD ± 3.1)	3.8 (SD ± 3.8)	3.3 (SD ± 3.1)	0.376
By osteotomy		0.3 (SD ± 0.8)	0.3 (SD ± 0.8)	0.3 (SD ± 0.8)	0.359 359

^a^AP: antiplatelet medication; ^b^VKA: vitamin K antagonist; ^c^Heparin: subcutaneous low molecular weight heparin (LMWH) or intravenous unfractionated heparin (UFH) ^d^DOAC: direct oral anticoagulant.

For the categorical data (sex, location, and medication), differences between the groups were analyzed using a Chi-square test or a Fisher’s Exact Test. The continuous data (age and median number of extractions) were analyzed using the Wilcoxon-Mann–Whitney test, as they did not follow a Gaussian distribution according to the Shapiro–Wilk test.

*P-values < 0.05 were considered statistically significant.

In the random test set of 400 procedures, which was given to the senior surgeon and the algorithms after their training, 17 procedures showed postoperative bleeding episodes (4.25%). All four algorithms outperformed the senior surgeon in terms of balanced accuracy, sensitivity, and specificity (Table 2), as well as a range of other key performance indicators (Suppl. Table 1).

**Table 2:** Performance of the prognostic models of the algorithms and the surgeon in the test set (*n* = 400)

Model/ Surgeon	Balanced Accuracy	Accuracy	Sensitivity	Specificity	AUC^a^	Brier Score
LR^b^	0.580	0.90 (CI^c^ 0.86–0.92)	0.235	0.924	0.624	0.041
RF^d^	0.591	0.81 (CI 0.77–0.85)	0.353	0.830	0.671	0.043
XGB^e^	0.608	0.84 (CI 0.80–0.88)	0.353	0.864	0.646	0.042
KNN^f^	0.616	0.70 (CI 0.65–0.74)	0.529	0.702	0.623	0.042
Senior Surgeon	0.527	0.74 (CI 0.69–0.78)	0.294	0.760	-	-

^a^AUC: area under the receiver operating characteristic curve; ^b^LR: logistic regression; ^c^CI: Confidence interval; ^d^RF: random forest; ^e^XGB: eXtreme gradient boost; ^f^KNN: K-nearest neighbors.

95% confidence intervals are provided for accuracy. No confidence interval is reported for balanced accuracy, as it is a composite metric whose uncertainty depends on both sensitivity and specificity, and no standard analytical method is established for its estimation.

**Fig. 2:**
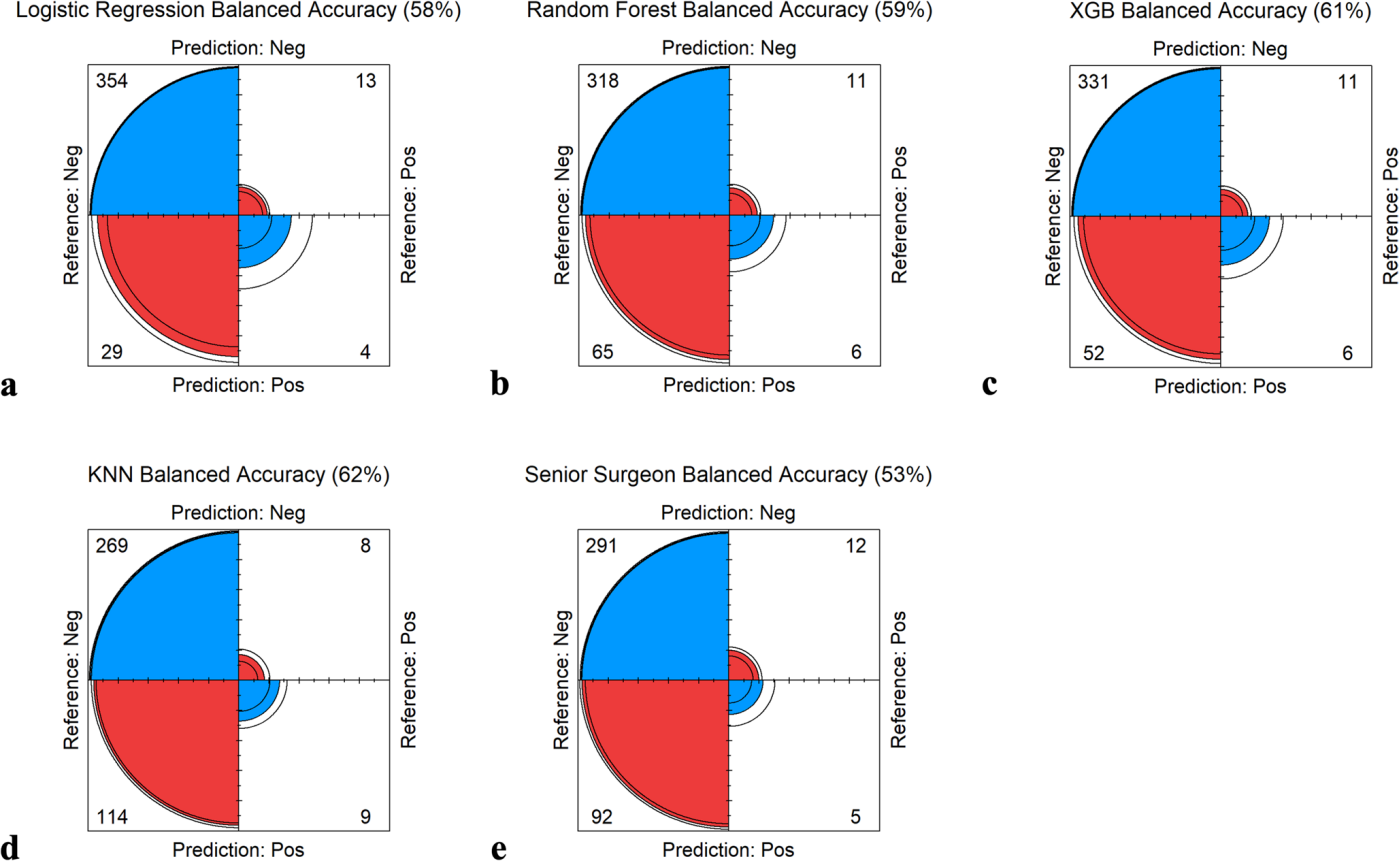
Accuracy of the different algorithms (a–d) and the senior surgeon (e) Classification outcomes for each diagnostic method. (a) Logistic Regression (LR): 4 true positives, 354 true negatives, 29 false positives, and 13 false negatives. (b) Random Forest (RF): 6 true positives, 318 true negatives, 65 false positives, and 11 false negatives. (c) eXtreme Gradient Boosting (XGB): 6 true positives, 331 true negatives, 52 false positives, and 11 false negatives. (d) K-Nearest Neighbors (KNN): 9 true positives, 269 true negatives, 114 false positives, and 8 false negatives. (e) Senior surgeon: 5 true positives, 291 true negatives, 92 false positives, and 12 false negatives

The LR had a balanced accuracy of 58% but identified only 4 of the 17 bleeding incidents. Although the surgeon’s balanced accuracy was lower (53%), he predicted 5 of the bleeding episodes but had a substantially higher share of false positive predictions (92 cases), as he predicted 97 bleeding incidents in total. The RF and XGB algorithms both identified 6 out of 17 postoperative bleeding incidents; however, the XGB algorithm was superior, as it had only 52 false positive results, compared to the RF algorithm, which predicted 71 bleeding incidents in total (65 false positives). KNN was the algorithm with the highest balanced accuracy (62%), identifying 9 out of 17 bleeding episodes. However, it also showed the highest rate of false positive predictions (114 cases) (Fig. 2).

## Discussion

Postoperative bleeding after oral surgery is a complication that has been evaluated in multiple studies [1, 3, 5, 6, 8, 9, 22]. Although the overall incidence is low, it can be a burden, especially for older and medically compromised patients. Hence, there have been attempts to assess individual risk to calculate the benefit of longer observation periods or a preventive inpatient setting [3, 23–25].

Previous studies have found that dual medication, age, osteotomies, and the number of removed teeth can influence postoperative bleeding risk after dental extractions [9, 17, 26, 27]. When conducting univariate analyses, as in this study’s descriptive statistics, only dual medication has been found to be a significant factor for bleeding risk. However, this kind of statistic cannot reveal multivariate interactions, nonlinear patterns, and other distribution specifics in the data. A few studies with large patient cohorts used LR analysis to control for variables such as medication, the extent of surgery, and patient-specific variables, such as age and sex [8, 9]. However, regression models cannot develop greater accuracy by performing multiple runs through the data and can be subject to spurious interpretations when there is multicollinearity between variables.

ML algorithms have shown advantages in other medical settings, such as the evaluation of dental X-rays or decision-making in the context of dental caries, as they are able to capture more complex patterns and can handle multicollinearity differently than traditional statistical models [14–16].

In this study, when using balanced accuracy as the evaluation metric, the ML algorithms XGB, KNN, and RF, as well as LR, outperformed a senior oral surgeon with more than 15 years of experience. This study’s results show that surgeons tend to overestimate the risk of bleeding, and that it can be overwhelming to integrate and weight many different parameters that have been shown to affect bleeding risk to some extent, into a single assessment. This is in line with a previous study that showed that a high share of patients who were preventively admitted to inpatient care were often not those in whom bleeding subsequently occurred [3]. Nevertheless, the German S3 guidelines recommend preventive hospitalization as an option for patients receiving dual or triple anticoagulation therapy [28].

KNN achieved the highest balanced accuracy (62%) and correctly identified 9 out of 17 bleeding episodes (sensitivity: 53%). However, these results also showed a share of false-negative predictions across all algorithms as well as in the surgeon’s evaluation.

The incidence of bleeding in this dataset was 4.35%, which lies below the incidence found in past studies [5, 9, 17, 20]. For example, Yanamoto et al. [5] reported a bleeding rate of 17.4%, Ueda et al. [9] found 19%, and other studies observed rates ranging from 10.4% to 23%, depending on patient population, antithrombotic medication, and procedure [17, 20].

One possible explanation for the lower incidence in our cohort is the standardized use of gelatin sponges in all procedures, which is known to significantly reduce bleeding events. Mahmoudi et al. [21], for instance, measured the average amount of blood absorbed by sterile gauze in a group treated with gelatin sponges compared to a control group and found significantly higher blood absorption in the control group. Svensson et al., who evaluated postoperative bleeding in patients on warfarin after dental extractions using an absorbable hemostatic gelatin sponge, reported a postoperative bleeding incidence of 4%, which is comparable to the incidence observed in this study [29]. This uniform application of local hemostatic measures may have acted as a confounding factor, lowering the overall bleeding risk and potentially making it more difficult for both the algorithms and the surgeon to accurately predict bleeding events. While this standardization ensures consistency across all cases and strengthens internal validity, it may limit the generalizability of the findings to clinical settings where such hemostatic measures are not routinely used. Consequently, an even larger sample size might have been necessary to detect subtle predictive patterns under these low-risk conditions.

In a medical setting, it is more important to identify actual bleeding episodes than to have a low rate of false positive results, which therefore makes KNN the safest prediction method. Nevertheless, its high rate of false bleeding predictions could be a cost factor resulting in unnecessary preventive costs. These would have been similarly high based on the senior surgeon’s inpatient care recommendations. Nevertheless, although XGB showed a lower balanced accuracy than KNN (61% vs. 62%), it was still superior to the senior surgeon (53%) and evaluated only 52 false positive (compared to 114 using KNN and 92 predicted by the surgeon), which makes it more cost-efficient with still high accuracy.

A limitation of this study is its retrospective, single-center design, which restricted the availability of certain clinical data. Consequently, the dataset did not include information about incisions, preoperative inflammation, or the experience level of the surgeons performing the extractions, all of which could potentially affect bleeding incidence [9, 30]. Furthermore, it must be considered that including a larger number of variables requires a correspondingly larger sample size to avoid overfitting and ensure robust model performance.

Another methodological constraint lies in the categorization of antithrombotic therapy. To preserve model generalizability and reduce multicollinearity, dual and triple therapies were not further subdivided into specific drug combinations (e.g., dual antiplatelet therapy vs. antiplatelet–anticoagulant regimens). While this approach supported model stability and interpretability, it limited analytical granularity and may have obscured differential bleeding risks associated with particular medication types.

Similarly, postoperative bleeding was assessed using a binary outcome (bleeding vs. no bleeding), without classification by clinical severity. Although distinctions between minor oozing and more significant events—such as bleeding requiring suturing or hospital-based intervention—would have added clinical depth, the low number of severe cases precluded reliable stratification. Introducing additional outcome categories in this context would have led to imbalanced subgroups and required either training multiple separate models or using multi-class classification approaches, which are not supported by all algorithms used in this study.

Therefore, a dichotomous definition was chosen to ensure consistent model training, maintain statistical power, and facilitate comparability across methods. Nonetheless, the lack of severity grading limits the clinical resolution of the results. Future studies with larger cohorts may enable more nuanced outcome definitions and a refined analysis of risk factors for clinically relevant bleeding.

As the KNN algorithm was able to identify four more bleeding incidents than the surgeon (+ 23.5%), this indicates that the model was able to pick up on patterns that were not consciously recognized by the surgeon. Advanced algorithms can therefore serve as valuable input for decision-making, especially for less experienced surgeons, due to their ability to consider complex patterns that are not salient or are hard to assess for the clinical practitioner.

However, the comparison of predictions to the assessment of a single clinician represents a potential source of bias—either positive or negative—and limits the generalizability of conclusions regarding the relative performance of machine learning models and clinical judgment.

Previous studies using artificial intelligence (AI) have already shown superiority to human approaches in other areas of dentistry: Bayrakdar et al. used a convolutional neural network algorithm for decision support in detecting caries and found higher accuracy using AI than when the dentists evaluated the X-rays [31], while Elgarba et al. compared an AI-driven implant placement tool and human intelligence and found the AI to perform expert-quality and clinically acceptable single-implant planning while being more time-efficient and consistent than the human approach [13]. Hence, especially in the diagnosis of X-rays, algorithms can be very useful and contribute to time and cost efficacy.

However, predictions based on clinical data seem more complex for ML algorithms, as studies that also used LR, RF, XGB, and KNN have shown. Long et al. evaluated the success of direct pulp capping [32], Cui et al. used ML for decision support concerning dental extractions [33], and Alshwayyat et al. evaluated survival predictions for mucoepidermoid carcinoma [34]. Based on the prediction complexity, the area under the receiver operating characteristic curve (AUC) values in these three studies were 0.86 [32], 0.97 [33], and 0.55 [34]. In this study, KNN achieved the highest balanced accuracy among the tested models, with an AUC of 0.62. This relatively modest discriminative performance suggests substantial heterogeneity within the dataset, likely due to unclear clinical patterns and the influence of unmeasured or unrecorded variables (e.g., incision technique, preoperative inflammation, or experience of the surgeon performing the procedure) affecting the risk of postoperative bleeding.

It is important to note that ML algorithms can also produce incorrect predictions, as physiological and pathological processes can be complex. Therefore, clinicians should never rely solely on the recommendations of ML or other AI models [35].

Particularly in older and medically compromised patients, postoperative decisions such as prolonged observation or preventive hospitalization often depend on non-quantifiable “soft” factors— including the patient’s home environment, level of independence, anxiety, and need for additional care. These aspects are typically difficult to capture through structured data alone and are best evaluated through clinical experience and personal judgment. While ML models can support clinicians by offering risk estimations based on structured, objective variables (“hard” data), they should be viewed as complementary tools. The final decision regarding postoperative risk management must always rely on a holistic assessment, integrating both data-driven insights and individual patient context.

Nevertheless, even with comprehensive modeling, certain bleeding episodes remained unpredictable. These cases may be related to factors that are not captured in structured clinical data, such as patient compliance, variability in surgical technique, accidental loosening of sutures, or other idiopathic causes. Such unpredictability highlights the limitations of current datasets and the complexity of real-world clinical scenarios.

Future studies should aim to incorporate a broader range of perioperative and follow-up variables, including qualitative or contextual information, to better reflect the nuanced factors influencing postoperative bleeding. In addition, larger and more diverse patient cohorts— ideally across multiple institutions— could help validate and generalize the performance of predictive models in everyday clinical practice.

## Conclusions

Preoperative prediction of bleeding episodes could contribute to better surgical planning, such as prolonged observation periods, inpatient treatment, or early day schedules for the procedures associated with a high risk.

To the best of our knowledge, this is the first study using an ML model in the context of complications after oral surgery. Even based on a sample size of 2000 procedures and a test set of 400 procedures, it outperformed an experienced human decision-maker. Connecting larger databases in future multicenter studies could lead to even better prediction of bleeding after extractions, contributing to better preventive care and cost efficiency.

## Supplementary Information

Below is the link to the electronic supplementary material.

Supplementary Material 1

## Data Availability

The datasets used and/or analyzed during the current study available from the corresponding author on reasonable request.
