# Supplementary material for: Evaluation of postoperative bleeding risk after dental extractions in patients on antithrombotic medication: A comparison of machine learning and clinical experience
Source: Clin Oral Investig. 2025 Oct 27;29(11):531. doi: 10.1007/s00784-025-06590-0 (PMC12554821; doi:10.1007/s00784-025-06590-0)
Supplement: Supplementary file 1 — Supplementary Material 1 [file 784_2025_6590_MOESM1_ESM.docx]

**Supplementary Table 1. Additional performance metrics of the prognostic models and the surgeon in the test set (n = 400)**

| Model/  Surgeon | Precision | Recall | F1 Score^a^ | PPV^b^ | NPV^c^ | Detection rate | Detection  Prevalence |
| --- | --- | --- | --- | --- | --- | --- | --- |
| LR^d^ | 0.121 | 0.235 | 0.160 | 0.121 | 0.965 | 0.010 | 0.083 |
| RF^e^ | 0.085 | 0.353 | 0.136 | 0.084 | 0.967 | 0.015 | 0.178 |
| XGB^f^ | 0.103 | 0.353 | 0.160 | 0.103 | 0.968 | 0.015 | 0.145 |
| KNN^g^ | 0.073 | 0.529 | 0.129 | 0.073 | 0.971 | 0.023 | 0.308 |
| Senior Surgeon | 0.052 | 0.294 | 0.088 | 0.051 | 0.960 | 0.013 | 0.243 |

^a^F1 Score: the harmonic mean of precision and recall; ^b^PPV: positive predictive value; ^c^NPV: negative predictive value; ^d^LR: logistic regression; ^e^RF: random forest; ^f^XGB: eXtreme gradient boost; ^g^KNN: K-nearest neighbors.
